# Quantification of Mechanical Factors in the Control of Weld Hot Cracking

**DOI:** 10.3390/ma19040712

**Published:** 2026-02-12

**Authors:** Wenda Wang, Shintaro Maeda, Kazuki Ikushima, Takahiro Osuki, Kazuhiro Ogawa, Hiroaki Mori, Masakazu Shibahara

**Affiliations:** 1 Graduate School of Engineering, Osaka Metropolitan University, Sakai 599-8531, Japan; su23524l@st.omu.ac.jp (W.W.);; 2 Nippon Steel Corporation, Amagasaki 660-0891, Japan; 3 Joining and Welding Research Institute, The University of Osaka, Ibaraki 567-0047, Japan; 4 Graduate School of Engineering, The University of Osaka, Suita 565-0871, Japan

**Keywords:** weld hot cracking, brittle temperature range (BTR), restraint coefficients, side-bead cracking test, critical curvature

## Abstract

Hot cracking in fully austenitic stainless steel welds is mainly caused by plastic strain accumulated while the weld cools through the brittle temperature range (BTR). This study proposes a simple mechanical model to estimate the plastic strain increment in the BTR and to clarify the effects of heat input and plate size (thickness and width). In the model, the BTR plastic strain increment is expressed as the sum of three terms: thermal contraction strain, bending strain due to a non-uniform temperature field, and an additional term caused by external restraint. Hot cracking is judged by whether the BTR plastic strain increment exceeds the critical strain. The model is applied to a restrained plate hot cracking test and a side-bead cracking test. For the side-bead test, we formulate the crack-driving bending moment per unit weld length and derive a simple relation between crack-tip curvature and local plate width. Using this relation, the critical-strain criterion is converted into a critical curvature. The critical curvature provides a practical index to compare cracking sensitivity for different geometries and heat inputs in welded structures.

## 1. Introduction

With the increasing size of chemical tankers and LNG carriers, cargo tanks and bulkhead structures require high corrosion resistance and fatigue strength, and austenitic stainless steels are widely used [1,2,3]. While these steels have excellent weldability and low-temperature toughness, they also have high thermal strain coefficients and low thermal conductivity. As a result, large tensile strains tend to develop during the cooling process of welding, and hot cracking (solidification cracking) can occur in the weld metal at the final stage of solidification [4,5]. Solidification cracking not only reduces the effective cross-sectional area of the joint but also decreases productivity due to increased inspection and repair work. Therefore, it is important to understand the mechanism of solidification cracking and quantitatively evaluate its susceptibility [6].

Since the pioneering work by Pellini [7], research on solidification cracking has established a framework for evaluating crack formation based on the relationship between the strain acting in the brittle temperature range (BTR), i.e., the temperature interval between the liquidus and solidus temperatures defined by the high-temperature ductility curve, and the critical strain of the material [8]. Singer and Prokhorov organized cracking susceptibility in terms of the relationship between alloy composition and the solid–liquid coexistence temperature range [8,9], while Matsuda and Senda developed the Trans-Varestraint test method and proposed the Critical Strain for Temperature Drop (CST) by combining BTR with critical strain [10]. These metallurgical approaches are effective for comparing material compositions and welding consumables, but they require specialized test equipment and complex specimen geometries, making it difficult to simply account for mechanical factors such as plate thickness, plate width, and restraint conditions in actual structures.

Against this background, self-restraint-type side-bead cracking tests, originating from the simple cracking test by Houldcroft, have been proposed and applied to evaluate solidification cracking susceptibility in various materials such as F82H steel and aluminum alloys [11,12,13]. Furthermore, studies combining side-bead cracking tests and finite element analysis have been conducted for austenitic stainless steel welds, and it has been shown that the initiation, propagation, and arrest behaviors of cracks can be reproduced using the BTR plastic strain increment [14,15]. However, these analyses require large model construction and computational cost, and have not been organized as a simple mechanical index for systematically examining plate thickness, plate width, and heat input conditions at the design stage. From a mechanical viewpoint, solidification cracking susceptibility has also been evaluated using strain- or strain-rate-based criteria obtained from thermo-mechanical analyses, where the accumulated deformation within a crack-susceptible temperature interval is compared with an empirically calibrated threshold for a given test configuration [14,15,16]. While such approaches can reproduce crack initiation and arrest behaviors, they often require case-by-case numerical evaluation and do not explicitly separate the respective contributions of thermal contraction, temperature-gradient-driven bending, and global restraint.The present study addresses this gap by providing a closed-form decomposition of the BTR plastic strain increment and by introducing a curvature-based representation for the side-bead configuration, thereby offering a design-oriented index for geometry and restraint effects.

In this study, we propose a simple mechanical model in which the BTR plastic strain increment is decomposed into thermal contraction, bending, and restraint components. The model is applied to restrained hot-cracking tests to evaluate the effects of plate thickness and heat input on solidification cracking susceptibility, and to side-bead cracking tests to interpret the plate-width dependence through a curvature-based formulation. For the side-bead test, the local rotation near the weld is approximated by beam theory, and a beam-theory-based formulation is presented to compute the curvature at crack initiation and relate it to the plate width at crack arrest. The applicability of this formulation is examined by comparison with our three-dimensional solidification cracking analysis. The material critical strain is then converted to a critical curvature, providing a design-oriented index that connects BTR-based metallurgical evaluation to geometry- and condition-dependent mechanical assessment. This unified formulation enables rapid parametric guidance without repeatedly performing full-scale three-dimensional finite element analysis.

## 2. A Simple Mechanical Model Based on the BTR Plastic Strain Increment

### 2.1. Overview of the Model

**Figure 1:**
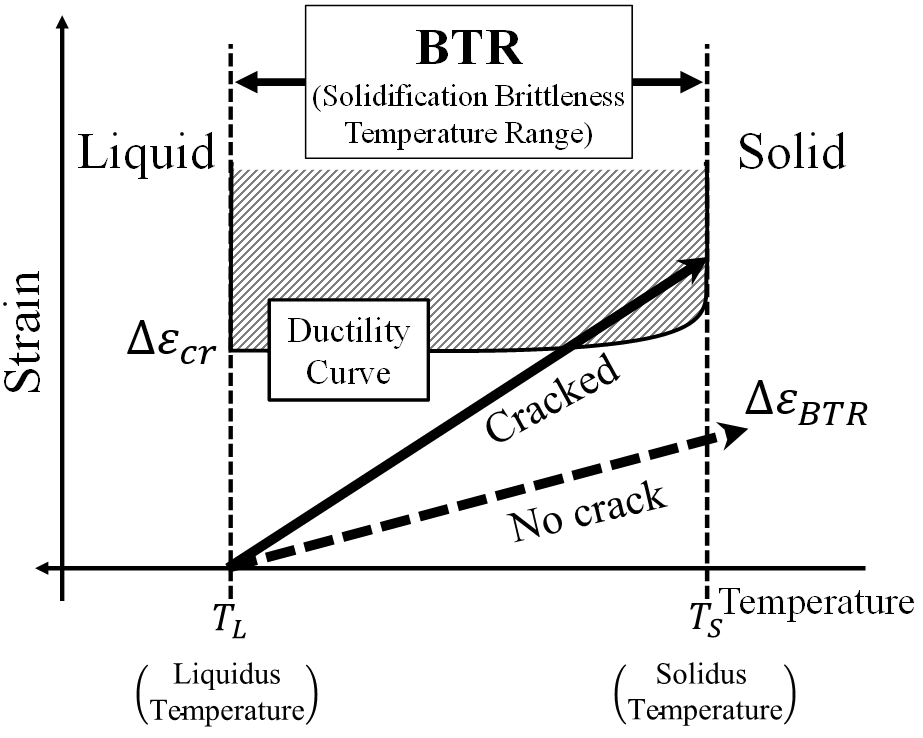
Schematic illustration of initiation of hot cracking.

As shown in Figure 1, this study focuses on the plastic strain increment that accumulates while the weld metal passes through the brittle temperature range (BTR), and evaluates weld hot cracking mechanically using a simple analytical model. The BTR is defined as the temperature interval from the liquidus temperature $T_{L}$ to the solidus temperature $T_{S}$. Because the ductility of the weld metal decreases significantly in this range, hot cracking is likely to occur when tensile strain concentrates in the transverse (plate-width) direction [15]. In the welding literature, it is widely accepted that weld hot cracking susceptibility can be assessed using strain-based criteria (critical strain/threshold strain concepts), and such criteria have been established and discussed in numerous studies including the Trans-Varestraint approach and the Threshold Strain Method [8,10,17].

In this study, weld hot cracking (solidification cracking in the weld metal during cooling through the BTR) is evaluated within a strain-based framework. For the transverse plastic strain, the BTR plastic strain increment $\Delta \varepsilon_{\mathrm{BTR}}$ is defined (during cooling through the BTR adopted in this study) as the difference between the plastic strain at the end of the BTR (at $T_{\mathrm{BTR},\mathrm{lower}}$) and that at the beginning of the BTR (at $T_{\mathrm{BTR},\mathrm{upper}}$) [15]:

(1) $$\Delta \varepsilon_{\mathrm{BTR}}=\varepsilon_{y}^{p}\left(T_{\mathrm{BTR},\mathrm{lower}}\right)-\varepsilon_{y}^{p}\left(T_{\mathrm{BTR},\mathrm{upper}}\right),$$

where $\varepsilon_{y}^{p}\left(T\right)$ is the transverse plastic strain. Using the effective critical threshold $\Delta \varepsilon_{\mathrm{cr}}$, hot cracking is judged by

(2) $$\Delta \varepsilon_{\mathrm{BTR}}>\Delta \varepsilon_{\mathrm{cr}}.$$

The critical threshold $\Delta \varepsilon_{\mathrm{cr}}$ is governed mainly by metallurgical factors such as alloy composition, solidification mode, and precipitation state, and can be calibrated using experimental methods such as the Trans-Varestraint test [10]. It should be noted that $\Delta \varepsilon_{\mathrm{cr}}$ may depend on welding conditions such as heat input and cooling rate through their effects on the strain rate during solidification [10,15]; therefore, when comparing different materials or welding conditions, $\Delta \varepsilon_{\mathrm{cr}}$ should be calibrated under consistent parameters. In contrast, the purpose of the present simple mechanical model is to evaluate analytically how deformation mode and restraint conditions affect $\Delta \varepsilon_{\mathrm{BTR}}$. Therefore, the same framework can be applied to other alloys by recalibrating the material-dependent parameter $\Delta \varepsilon_{\mathrm{cr}}$ (and, if necessary, the adopted BTR bounds) under consistent welding conditions.

**Figure 2:**
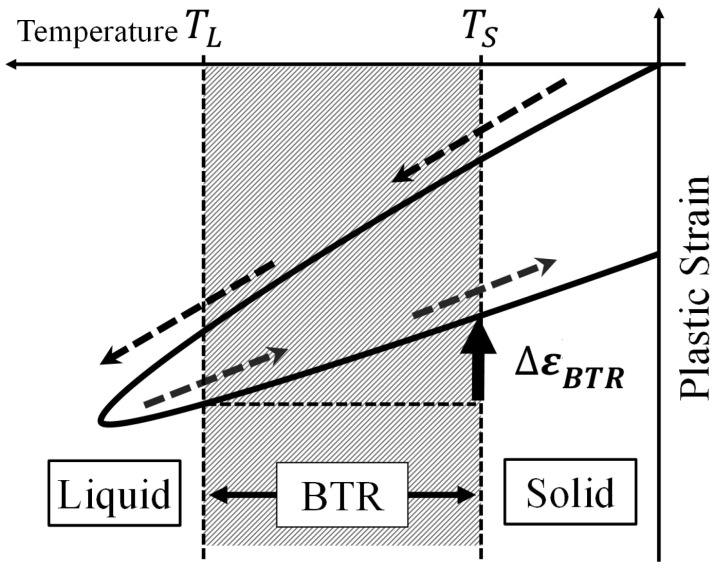
Schematic illustration of plastic strain increment in BTR during cooling.

Figure 2 illustrates the concept of the BTR-based index. Plastic strain increases while the weld cools through the BTR from $T_{L}$ to $T_{S}$ ($T_{L}\to T_{S}$), and hot cracking is assumed to occur when the increment exceeds $\Delta \varepsilon_{\mathrm{cr}}$. Modeling the weld transverse section as a beam, the transverse strain accumulated during the BTR is decomposed into three components: (i) strain due to thermal contraction $\Delta \varepsilon_{T}$, (ii) strain due to bending deformation $\Delta \varepsilon_{B}$, and (iii) an additional strain produced by external restraint $\Delta \varepsilon_{f}$. Then,

(3) $$\Delta \varepsilon_{\mathrm{BTR}}=\Delta \varepsilon_{T}+\Delta \varepsilon_{B}+\Delta \varepsilon_{f}.$$

Here, $\Delta \varepsilon_{f}$ represents the additional transverse strain increment induced by global restraint of the plate. In the present framework, it should be interpreted as the strain increment associated with the mismatch between the deformation that would occur under a free (low-restraint) condition during BTR cooling (free shrinkage and free angular distortion) and the deformation actually allowed under restraint. This term is distinguished from the bending component $\Delta \varepsilon_{B}$, which is driven by the non-uniform temperature distribution through the thickness, whereas $\Delta \varepsilon_{f}$ arises from suppression of the overall plate deformation by the surrounding structural restraint.

From an engineering viewpoint, $\Delta \varepsilon_{f}$ can be approximated if the transient angular distortion (or global out-of-plane deformation) under a free-rotation condition, $\theta_{\mathrm{free}}$, and that in the actual welded structure, θ, are available (measured or computed with a simplified structural stiffness/compliance model). The difference $\left(\theta_{\mathrm{free}}-\theta \right)$ quantifies the restraint-induced reduction of global deformation and can be converted into an equivalent additional transverse strain increment at the weld line using the same beam kinematics adopted in the present model.

**Figure 3:**
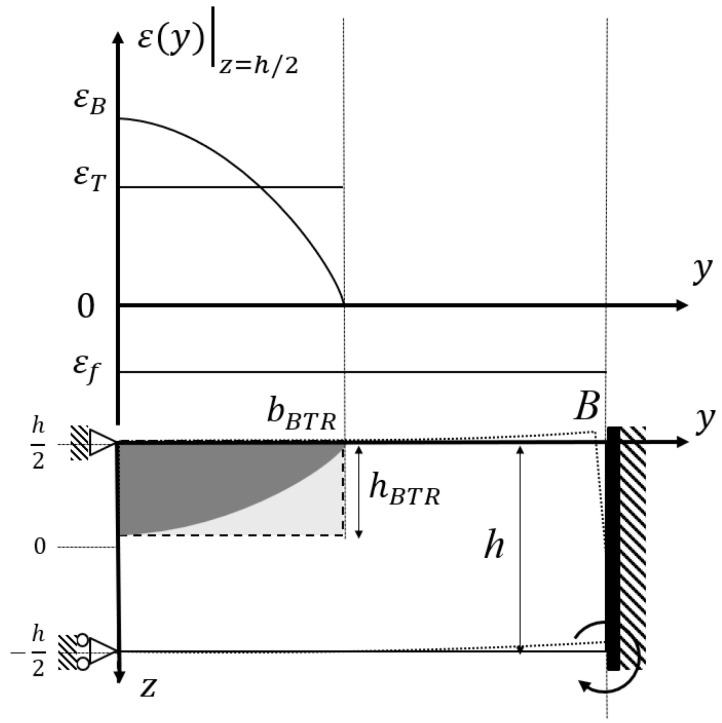
Schematic beam model of a weld cross-section and decomposition of plastic strain components.

This relation provides the basic framework to organize the strain generated in the BTR in terms of three mechanical factors: thermal contraction, thermally induced bending, and external restraint. Figure 3 schematically shows how these three components arise in the beam model of the weld transverse section. The modeling of each component is described below.

### 2.2. Components of the Plastic Strain in the BTR

#### 2.2.1. Thermal Contraction Strain $\Delta \varepsilon_{T}$

As the temperature decreases during the BTR, the weld metal contracts in the transverse direction. Assuming that the temperature is uniform through the thickness, the thermal contraction strain generated within the BTR is approximated by

(4) $$\Delta \varepsilon_{T}≃\alpha \left(T_{L}-T_{S}\right),$$

where α is the coefficient of linear thermal strain. In practice, the through-thickness temperature distribution changes with time; however, when the change in the thickness-averaged temperature is dominant, Equation (4) provides a representative estimate of the thermal contraction strain accumulated while cooling through the BTR [18].

#### 2.2.2. Bending Strain $\Delta \varepsilon_{B}$

When the temperature distribution through the thickness is non-uniform, a thermally induced bending moment develops in the weld transverse section, and an additional transverse bending strain is applied to the weld metal. Based on beam theory, the transverse strain can be decomposed into a membrane component (section-average) and a curvature component. The strain due to bending is written as

(5) $$\varepsilon_{B}\left(z\right)=\kappa z,$$

where κ is the curvature and *z* is the thickness coordinate measured from the neutral axis. For the bending moment $M_{B}$,

(6) $$\kappa =\frac{M_{B}}{EI},$$

where *E* is Young’s modulus and *I* is the second moment of area of the section. Therefore, the bending strain at the weld-side surface (z=h/2, where *h* is the plate thickness) is $\varepsilon_{B}\left(h/2\right)=\kappa h/2$, and its increment within the BTR is expressed as

(7) $$\Delta \varepsilon_{B}=\frac{h}{2EI}\Delta M_{B}=\frac{h}{2EI}\left[\int_{-\frac{h}{2}}^{\frac{h}{2}}E\alpha T_{L}\left(z\right)\left(z-\frac{h}{2}\right)\;dz-\int_{-\frac{h}{2}}^{\frac{h}{2}}E\alpha T_{S}\left(z\right)\left(z-\frac{h}{2}\right)\;dz\right].$$

The bending component depends mainly on the through-thickness temperature gradient and the plate thickness *h*, and it develops locally near the weld (see Figure 3).

#### 2.2.3. Strain Produced by External Restraint $\Delta \varepsilon_{f}$

The discussion above assumes that the weld can rotate (bend) freely without external restraint. In real structures, however, rotational deformation is often restricted by fillet welds, fixtures, and surrounding structural members. In the free state, rotation occurs according to the temperature gradient; when rotation is suppressed by external restraint, an additional tensile strain arises in the transverse direction. This additional strain is denoted as $\Delta \varepsilon_{f}$.

In modeling the transverse section including the weld line as a beam, let $\theta_{\mathrm{free}}$ be the free bending rotation angle generated in the BTR. When external restraint enforces θ=0, an additional restraint moment $\Delta M_{f}$ is induced to cancel the free rotation. The additional curvature $\kappa_{f}$ is

(8) $$\kappa_{f}=\frac{\Delta M_{f}}{EI},$$

and the corresponding strain at the plate surfaces is

(9) $$\varepsilon_{f}\left(z=\pm \frac{h}{2}\right)=\kappa_{f}\frac{h}{2}=\frac{\Delta M_{f}\;h}{2EI}.$$

By evaluating $\Delta M_{f}$ so that the difference between the free rotation and the restraint condition satisfies

(10) $$\theta_{\mathrm{free}}+\theta_{f}=0,\;\theta_{f}=-\int_{0}^{B}\kappa_{f}\left(y\right)\;dy.$$

the strain increment $\Delta \varepsilon_{f}$ can be determined. The magnitude of $\Delta \varepsilon_{f}$ depends on the stiffness of surrounding structures and the restraint conditions; even for the same welding condition and plate thickness, stronger restraint increases $\Delta \varepsilon_{f}$ and raises the risk of weld hot cracking. Conversely, it may be possible to reduce $\Delta \varepsilon_{f}$ and suppress cracking by adopting fixture arrangements and welding sequences that relax restraint.

### 2.3. Evaluation of the Temperature Field Using an Instantaneous Line Heat Source Solution

To quantify the three strain components described above, it is necessary to evaluate the through-thickness temperature distribution T(r,t) during the BTR passage. In this study, the temperature variation along the weld line is assumed to be relatively gradual. Therefore, we focus on a transverse section normal to the weld line and approximate the temperature field based on an instantaneous line heat source solution.

We consider an orthogonal coordinate system with the weld direction as the *x*-axis, the plate-width direction as the *y*-axis, and the thickness direction as the *z*-axis. At a given position on the weld line, a line heat source is assumed to act at time t=0. For an infinite body, the temperature rise ΔT is given by [19]

(11) $$\Delta T\left(r,t\right)=\frac{\eta q}{4\pi \lambda t}\exp\left(-\frac{r^{2}}{4\alpha t}\right),$$

where $r^{2}=y^{2}+z^{2}$, η is the heat efficiency, *q* is the heat input per unit length, λ is the thermal conductivity, and α=λ/(ρc) is the thermal diffusivity, with ρ being the density and *c* the specific heat.

In actual welding, the plate thickness is finite and heat losses due to convection and radiation exist at the surfaces. Therefore, an approximate solution for a finite plate can be constructed by introducing image heat sources and adding surface heat-transfer boundary conditions [20]. From the resulting temperature field T(r,t), the through-thickness temperature distribution during the time interval when the material cools through the BTR range (from $T_{L}$ to $T_{S}$, $T_{L}\to T_{S}$) is extracted and used as input to the beam model described in the previous subsections.

### 2.4. Remarks on Restraint Boundary Conditions

**Figure 4:**
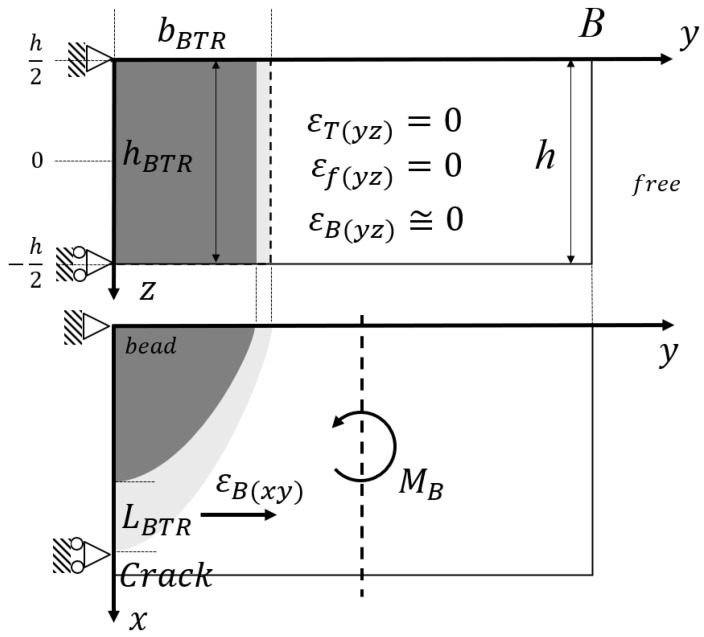
Schematic of the no-external-restraint limit and the crack-driving mechanism in the side-bead test.

In this study, the BTR plastic strain increment $\Delta \varepsilon_{\mathrm{BTR}}$ is used as the mechanical indicator for weld hot cracking, and crack initiation is judged based on Equations (2) and (3). Here, $\Delta \varepsilon_{T}$ is the thermal contraction (membrane) component due to the temperature drop in the BTR, $\Delta \varepsilon_{B}$ is the bending component caused by non-uniform temperature distribution, and $\Delta \varepsilon_{f}$ is the restraint component that arises when free rotation is suppressed by external restraint. In this subsection, the restrained hot cracking test (Figure 3) and the side-bead cracking test (Figure 4) are positioned as two representative cases in the common framework, where the dominant term changes depending on boundary conditions.

First, the restrained hot cracking test (Figure 3) represents a strongly restrained condition due to surrounding structures. As shown in Figure 3, the transverse plastic strain increment in the BTR region (characterized by $b_{\mathrm{BTR}}$ and $h_{\mathrm{BTR}}$) includes not only $\Delta \varepsilon_{T}$ (thermal contraction) and $\Delta \varepsilon_{B}$ (free bending), but also a significant $\Delta \varepsilon_{f}$ term associated with restraint reactions caused by suppression of rotational deformation. Therefore, $\Delta \varepsilon_{f}$ cannot be neglected in this test, and the relative contributions of $\Delta \varepsilon_{B}$ (bending) and $\Delta \varepsilon_{f}$ (restraint) change with plate thickness and restraint conditions, leading to thickness-dependent cracking susceptibility.

In contrast, the side-bead cracking test (Figure 4) is treated as a case with very small external restraint, and the restraint component can be approximated as $\Delta \varepsilon_{f}≃0$. Furthermore, as shown in Section 4, for the heat input conditions considered in this study, the temperature distribution relevant to the BTR region can be organized as an approximation depending only on the plate-width coordinate *y*. Therefore, among the components represented on the yz section (plate width–thickness section), the contribution to the tensile plastic strain increment in the *y* direction evaluated in the BTR is not dominant. This corresponds to the assumptions in Figure 4: $\varepsilon_{T}\left(yz\right)≃0$, $\varepsilon_{f}\left(yz\right)≃0$, and $\varepsilon_{B}\left(yz\right)≃0$. Under these assumptions, crack driving is governed mainly by the in-plane (the xy plane) bending moment $M_{B}$, and the crack propagation and arrest behaviors are controlled through the induced bending strain $\varepsilon_{B}\left(xy\right)$ in the BTR region.

In summary, the restrained hot cracking test (Figure 3) and the side-bead cracking test (Figure 4) share the common cracking criterion $\Delta \varepsilon_{\mathrm{BTR}}>\Delta \varepsilon_{\mathrm{cr}}$, while they can be organized consistently as two representative cases where the dominance of $\Delta \varepsilon_{B}$ and $\Delta \varepsilon_{f}$ switches depending on the strength of external restraint and the treatment of the temperature field. This discussion is intended for conditions where the evaluation location is sufficiently far from bead start/stop regions so that the thermal cycle can be regarded as quasi-steady along the weld line. In addition, the derivations assume small out-of-plane deflection/rotation; for the side-bead test, the curvature-based treatment becomes more reliable when the penetration depth is sufficiently large relative to the plate thickness, because the influence of the through-thickness temperature gradient on the local bending response is reduced.

## 3. Verification of the Simple Mechanical Model Using Restrained Hot Cracking Tests

In this section, the simple mechanical model based on the BTR plastic strain increment developed in Section 2 is applied to a restrained hot cracking test using a flat plate restrained on all sides by fillet welds. The effect of plate thickness on weld hot cracking susceptibility is examined. First, the specimen configuration and welding conditions are described, followed by the input conditions and analysis procedure for the simple mechanical model.

### 3.1. Analysis Model and Conditions

**Figure 5:**
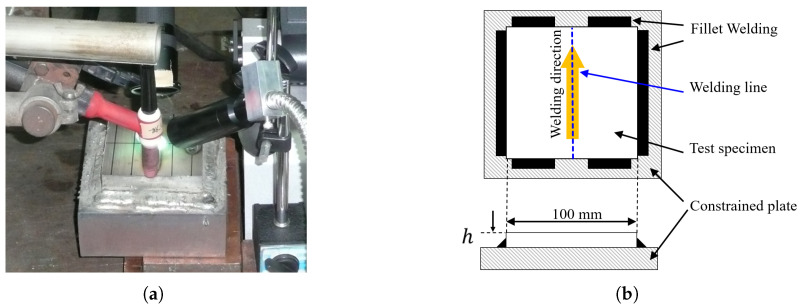
Schematic illustration of constrained hot cracking test specimen. (**a**) On-site experiment photo. (**b**) Illustrative configuration.

Figure 5 shows a schematic of the restrained hot cracking test. The test specimen is a flat plate measuring 100 mm in length and 100 mm in width, with plate thickness *h* varied in the range from 4 to 30 mm. The four edges of the specimen are joined to restraint plates of the same material by fillet welds, so that the central region of the specimen is subjected to strong external restraint from the surroundings. A single-pass bead-on-plate weld is applied along the longitudinal direction at the center of the specimen, and the occurrence of hot cracking in the central weld metal is observed.

The test material is based on a fully austenitic stainless steel SUS310, in which the solidification cracking susceptibility is controlled by adjusting the phosphorus (P) content. The contents of impurity elements such as C, S, and N are kept low, and the weld hot cracking susceptibility is primarily varied by the P content. The representative chemical composition is shown in Table 1, and the restrained hot cracking tests conducted in this section target material with a P content of approximately 0.1%, which exhibits high weld hot cracking susceptibility. Welding is performed by direct current TIG welding with uniform heat input conditions achieved by maintaining constant current, voltage, and welding speed. The representative welding conditions are shown in Table 2, and the heat input per unit length *Q* is given by Q=ηVI/v, where *V* is the arc voltage, *I* is the welding current, and *v* is the welding speed. By keeping the restraint and heat input conditions constant and parametrically varying only the plate thickness *h*, the effect of plate thickness as a mechanical factor on weld hot cracking susceptibility is extracted.

For the SUS310S(A) material with 0.1% P content, the critical strain for hot cracking judgment was treated as an effective threshold and calibrated as $\Delta \varepsilon_{\mathrm{cr}}\approx (2.0$–$2.5)\;\times \;10^{-3}$ (i.e., 0.20–0.25%) using side-bead cracking tests combined with finite element analysis (details in Section 4). Because this criterion can vary depending on welding conditions and the material state, $\Delta \varepsilon_{\mathrm{cr}}$ is treated here as a representative value for the present investigation. Notably, its magnitude is within a reasonable range when compared with the critical/threshold strain reported for SUS310S in previous work (TSSC ≈0.11%) [17]. The BTR (brittle temperature range) was defined as 1165–1400 °C based on high-temperature ductility tests [21]. This critical strain should be regarded as a representative value for the present welding condition and BTR definition.

**Figure 6:**
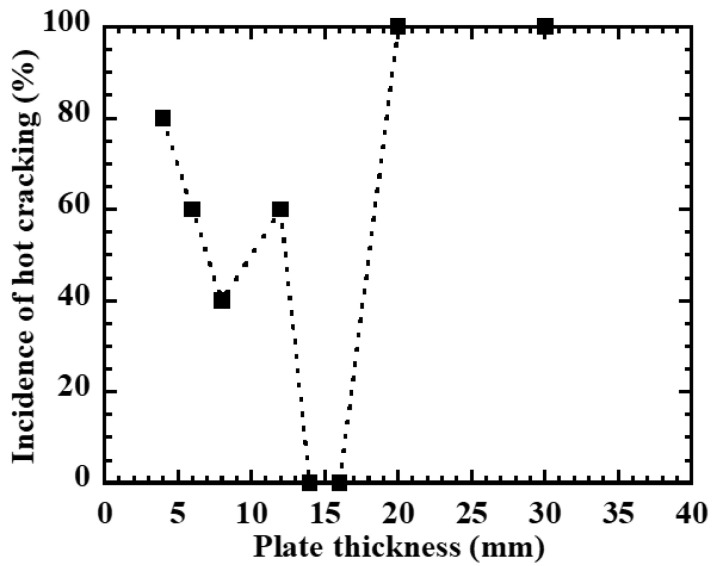
Incidence of hot cracking as a function of plate thickness in constrained plate test; data points are connected by dashed lines to indicate the general trend.

Multiple test specimens were prepared for each plate thickness condition. After welding completion and cooling to room temperature, macroscopic observations were conducted on cross-sections perpendicular to the weld line. When cracks propagating inward from the fusion boundary were clearly observed at the center of the weld metal, the specimen was judged as “cracked”; otherwise, it was judged as “crack-free”. When *n* specimens were welded at a given plate thickness condition and $n_{\mathrm{crack}}$ of them exhibited weld hot cracking, the cracking incidence rate $I_{\mathrm{cr}}$ was defined as

(12) $$I_{\mathrm{cr}}=\frac{n_{\mathrm{crack}}}{n}\times 100\;\left(\%\right)$$

and the relationship between plate thickness *h* and cracking incidence rate $I_{\mathrm{cr}}$ was determined. Figure 6 shows the results. In the thin plate region with thickness 4–8 mm, the cracking incidence rate is high, while in the region around 12–14 mm thickness, cracking hardly occurs. On the other hand, in the thick plate region exceeding 20 mm, the cracking incidence rate increases again, indicating that the weld hot cracking susceptibility changes non-monotonically with plate thickness.

### 3.2. Comparison of Restrained Hot Cracking Test Results with Simplified Mechanical Model and FEM Analysis

In this section, the relationship between plate thickness and cracking incidence rate in the restrained hot cracking test is compared with the results of three-dimensional finite element analysis and the simplified mechanical model developed in Section 2, and the plate thickness dependency is examined from the viewpoint of plastic strain within the BTR.

**Figure 7:**
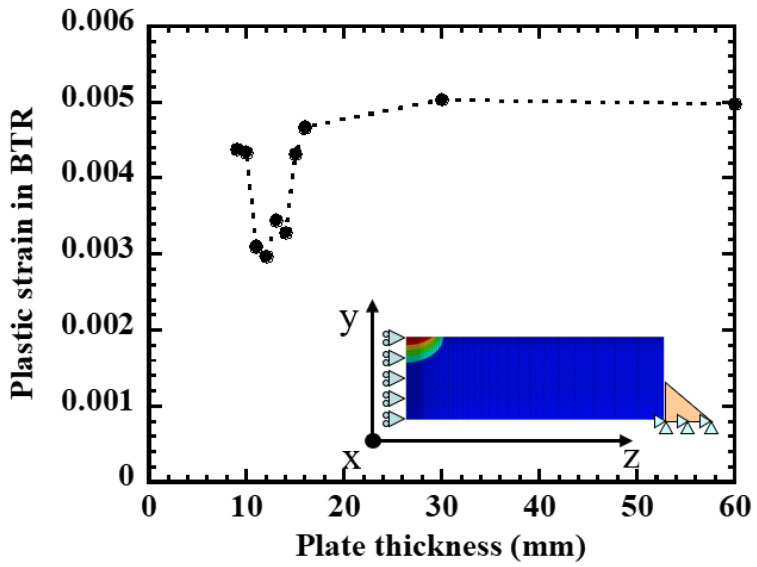
Plastic strain accumulated in the BTR at the weld center by FEM hot-cracking analysis; dashed lines are used to indicate trend.

First, three-dimensional finite element analysis was performed for the same geometry and welding conditions as the restrained hot cracking test, and the BTR plastic strain increment at the center of the weld metal was evaluated (Figure 7). The hot cracking analysis method proposed by Maeda [15] was adapted for the analysis procedure. In this method, the temperature history of each element is obtained by welding heat conduction analysis, and the stress–strain history is calculated by temperature–stress coupled analysis considering the temperature-dependent material properties of SUS310S(A). The BTR is defined as the temperature range between the liquidus temperature $T_{L}$ and the solidus temperature $T_{S}$, and the BTR plastic strain increment $\Delta \varepsilon_{\mathrm{BTR}}^{\mathrm{FEM}}$ is obtained by time-integrating the plastic strain accumulated in elements near the fusion boundary while passing through this temperature range. When $\Delta \varepsilon_{\mathrm{BTR}}^{\mathrm{FEM}}$ is organized by varying the plate thickness in the range of 4–50 mm, a non-monotonic distribution is obtained in which the value is large on both the thin and thick plate sides and minimum in the intermediate plate thickness range (approximately 12–14 mm). The plate thickness range where the cracking incidence rate shown in Figure 6 is lowest and the plate thickness range where $\Delta \varepsilon_{\mathrm{BTR}}^{\mathrm{FEM}}$ is minimum are approximately consistent, confirming that the plastic strain within the BTR is also minimized under plate thickness conditions where weld hot cracking is less likely to occur.

Next, the evaluation results using the simplified mechanical model introduced in Section 2 are described. The BTR (brittle temperature range during solidification) was given as a temperature range of approximately 1165–1400 °C for the 0.1% P material based on the liquidus temperature $T_{L}$ and solidus temperature $T_{S}$ obtained from high-temperature ductility tests [21]. A coordinate system was established with the *y*-axis in the direction perpendicular to the weld line and the *z*-axis in the plate thickness direction, and the temperature field T(r,t) was evaluated using the instantaneous line heat source solution for a representative cross-section on the weld line. The temperature distribution in the plate thickness direction T(r,t) during the period $t_{L}$ to $t_{S}$ passing through the BTR was extracted and input into the beam model shown in Section 2 to calculate the strain due to thermal contraction $\Delta \varepsilon_{T}$, the strain due to bending deformation $\Delta \varepsilon_{B}$, and the strain generated by external restraint $\Delta \varepsilon_{f}$ under perfectly restrained conditions.

**Figure 8:**
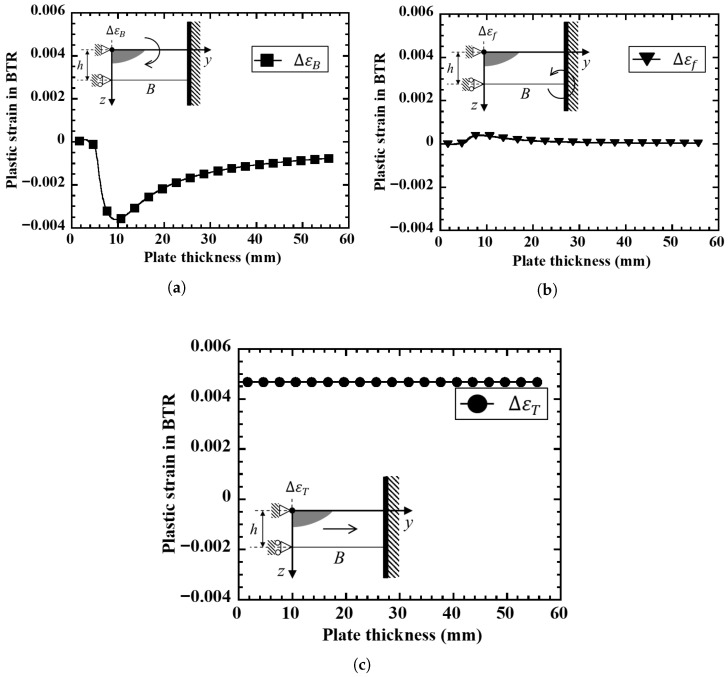
Plate thickness dependence of each plastic strain component in BTR calculated by the mechanical model. (**a**) Bending deformation. (**b**) External restraint. (**c**) Thermal contraction.

Figure 8 shows the results of calculating the plastic strain within the BTR separated into three components: thermal contraction strain $\Delta \varepsilon_{T}$, bending strain $\Delta \varepsilon_{B}$, and restraint strain $\Delta \varepsilon_{f}$ under the restrained hot cracking test conditions (plate width B=50 mm, heat input Q=945 J/mm). The thermal contraction strain $\Delta \varepsilon_{T}$ is almost constant regardless of plate thickness and is mainly governed by the heat input and the temperature range of the BTR. On the other hand, the bending strain $\Delta \varepsilon_{B}$ is large on the thin plate side and decreases sharply with increasing plate thickness initially, but shows a minimum value at around 10 mm plate thickness and then increases gradually thereafter. This is because in thin plates, the cross-sectional rigidity is small against the bending moment caused by the temperature gradient in the plate thickness direction, resulting in large bending strains on the top and bottom surfaces, whereas as the plate thickness increases, the second moment of area increases and bending deformation is suppressed even with the same temperature gradient. For engineering practice, $\Delta \varepsilon_{f}$ may be approximated from the difference in transient angular distortion at the weld line between a virtually free-rotation condition and the actual restrained structure, i.e., $\left(\theta_{\mathrm{free}}-\theta_{\mathrm{act}}\right)$, using the same beam kinematics adopted in this model. In practice, $\theta_{\mathrm{free}}$ may be obtained from a simplified local model with free-rotation boundary conditions, while $\theta_{\mathrm{act}}$ may be estimated from a coarse global FE model of the structure or fixture, or, where available, from in situ angular distortion measurements.

**Figure 9:**
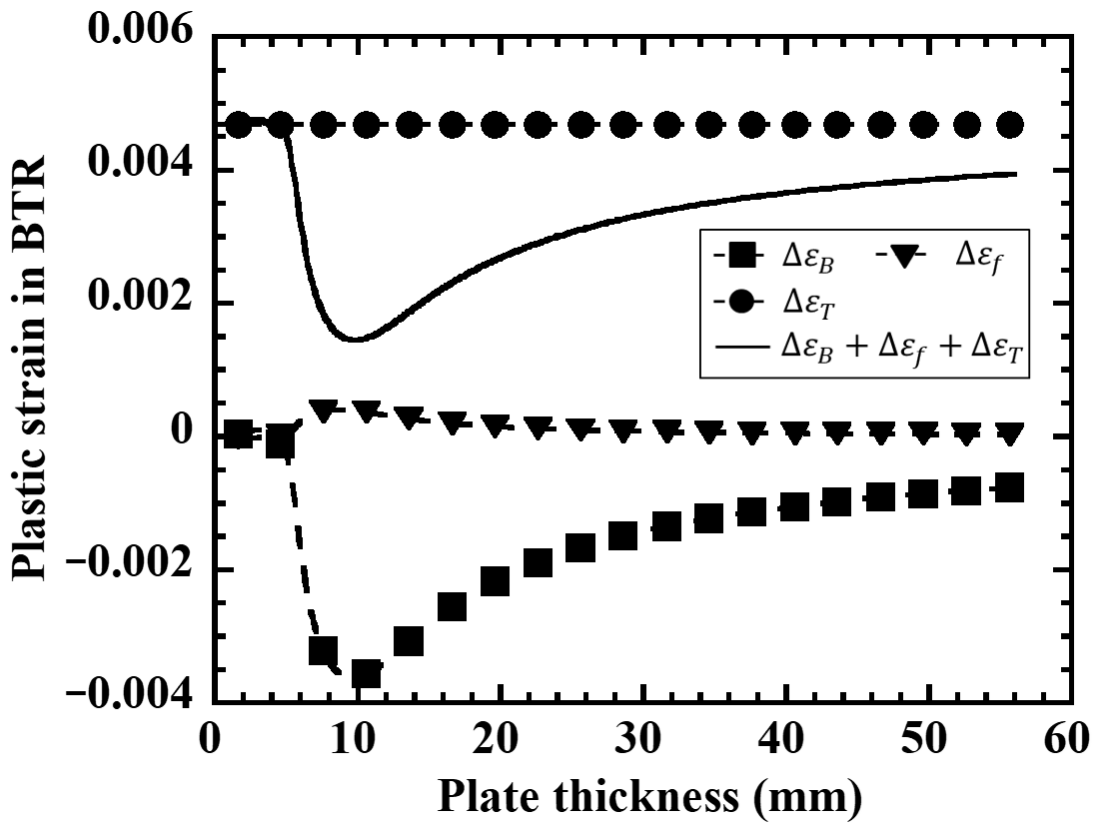
Plastic strain in BTR as a function of plate thickness calculated by the mechanical model.

The plate thickness dependence of the BTR plastic strain increment $\Delta \varepsilon_{\mathrm{BTR}}$ obtained as the sum of these three components is shown in Figure 9. On the thin plate side, $\Delta \varepsilon_{\mathrm{BTR}}$ is large due to the contribution of $\Delta \varepsilon_{B}$, takes a minimum value once in the intermediate plate thickness range, and then increases again on the thick plate side due to the contribution of $\Delta \varepsilon_{f}$, resulting in a non-monotonic distribution. The position where $\Delta \varepsilon_{\mathrm{BTR}}$ is minimum at around 12–14 mm plate thickness approximately coincides with the plate thickness range where the cracking incidence rate is lowest in Figure 6 and the plate thickness range where $\Delta \varepsilon_{\mathrm{BTR}}^{\mathrm{FEM}}$ is minimum in Figure 7. Here, if the cracking occurrence condition is defined as $\Delta \varepsilon_{\mathrm{BTR}}>\Delta \varepsilon_{\mathrm{cr}}$ using a material-specific critical strain $\Delta \varepsilon_{\mathrm{cr}}$, it can be interpreted that in the intermediate plate thickness range, $\Delta \varepsilon_{\mathrm{BTR}}$ falls below the critical value and cracking is less likely to occur, while on the thin and thick plate sides, $\Delta \varepsilon_{\mathrm{BTR}}$ exceeds the critical value due to the increase in either mechanical component ($\Delta \varepsilon_{B}$ or $\Delta \varepsilon_{f}$), resulting in high crack susceptibility. A discrepancy in the absolute values between Figure 7 and Figure 9 is observed, which can be attributed to the use of representative material properties in the BTR and the associated simplifications in the analytical formulation, whereas the qualitative trends remain consistent.

**Figure 10:**
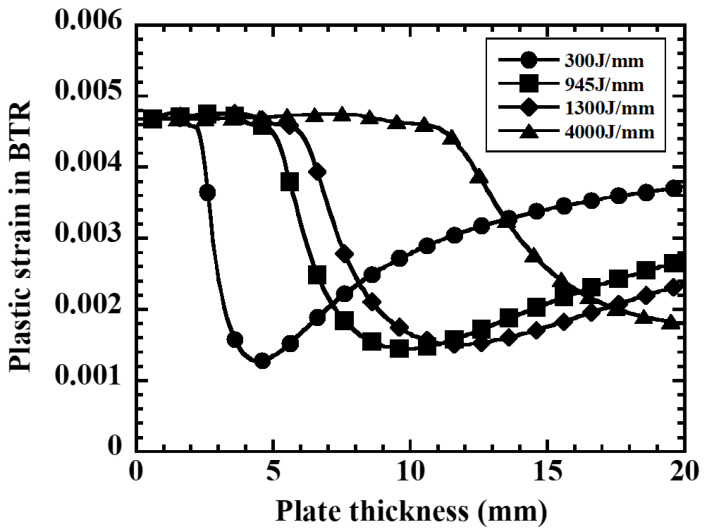
Effect of heat input on plastic strain in BTR and plate thickness to avoid hot cracking.

Furthermore, Figure 10 shows the results of examining the effect of heat input using the simplified mechanical model. When $\Delta \varepsilon_{\mathrm{BTR}}$ is calculated with heat inputs of *Q* = 300, 945, 1300, 4000 J/mm, the curves all show a non-monotonic distribution with respect to plate thickness, and there exists a plate thickness where $\Delta \varepsilon_{\mathrm{BTR}}$ is minimum for each curve. As the heat input increases, the entire curve shifts upward. This result indicates that not only plate thickness selection but also heat input control (such as multi-pass welding) is important for reducing weld hot cracking susceptibility.

From the above results, it can be seen that the experimental results of the restrained hot cracking test, the BTR plastic strain increment by three-dimensional FEM analysis based on the method of Maeda et al., and the evaluation results by the simplified mechanical model all show plate thickness and heat input dependencies in which the crack susceptibility is lowest in the intermediate plate thickness range and higher on the thin and thick plate sides. The present simplified mechanical model, which evaluates the BTR plastic strain increment by decomposing it into three components—thermal contraction strain increment, bending strain increment, and restraint strain increment—is effective as a mechanical approximation consistent with the detailed FEM method of Maeda et al., and can be used as an indicator when examining plate thickness and heat input conditions at the design stage. In Section 4, the same framework will be applied to the side-bead cracking test, and the evaluation of solidification cracking susceptibility based on plate width and curvature will be examined.

## 4. Hot Cracking Analysis in Side-Bead Cracking Test

In this section, the simplified mechanical model based on plastic strain within the BTR developed in Section 2 is applied to the side-bead cracking test with constant plate thickness and varying plate width, and the crack initiation, propagation, and arrest behaviors are examined from the viewpoint of plate width. First, the relationship between the curvature at the crack initiation location and the width at the crack arrest position is derived using beam theory, and the continuity with the plate thickness dependency model in Section 3 is clarified. Next, the side-bead cracking test is simulated using a three-dimensional thermo-elastic-plastic analysis model, and crack judgments based on BTR plastic strain increment and crack arrest width are obtained. Finally, using the curvature–plate width relationship diagram obtained from the theoretical equations, the analysis results are compared with the experimental results of the side-bead cracking test and the susceptibility evaluation by previous side-bead cracking tests, and the validity of this mechanical model is discussed.

### 4.1. Mechanical Model for Side-Bead Cracking Test

**Figure 11:**
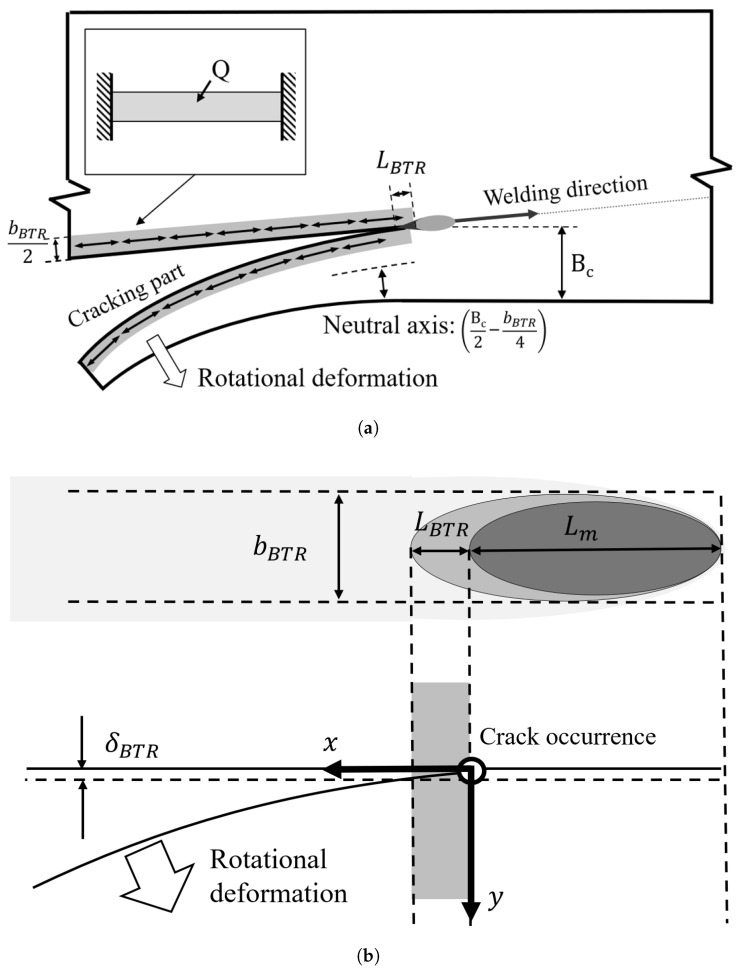
Schematic configuration of side-bead cracking test specimen. (**a**) Rotational distortion. (**b**) Plastic strain increment in BTR.

In the side-bead cracking test, bead-on-plate welding is performed along the vicinity of one edge of a thin plate with constant thickness, and the specimen is machined so that the plate width changes in the direction perpendicular to the weld line. Figure 11 shows a schematic of the side-bead cracking test specimen targeted in this study. The plate width B(x) is designed to increase or decrease linearly along the longitudinal direction *x*. The weld line is placed at a constant distance inside from the plate edge, and crack initiation behavior is observed at the center of the weld metal. In the side-bead cracking test, external restraint is small, and the strain driving within the BTR (during cooling) appears mainly as bending deformation in the plate width direction. In this section, based on the unified description used in Section 2, the heat input *Q*, resultant force *F*, and bending moment *M* are all defined as quantities per unit length on the weld line.

The temperature-drop magnitude across the BTR during cooling is defined as

(13) $$T_{\mathrm{BTR}}\equiv T_{L}-T_{S}$$

In the present configuration, the thermal cycle near the weld is treated as quasi-steady along the weld-line direction. In the side-bead model, the temperature field relevant to the local deformation is represented on a representative cross-section perpendicular to the weld line, because the bending driver is the non-uniform transverse thermal contraction during cooling across the BTR, i.e., the deviation component from the cross-sectional average. At this time, during cooling across the BTR, the thermal shrinkage strain increment is given by $\alpha T_{\mathrm{BTR}}$, and the deviation component excluding the cross-sectional average becomes the bending driver. Letting $B_{c}$ be the plate width at the crack arrest position and *h* be the plate thickness, the bending moment increment per unit length within the BTR is expressed as a unified equation:

(14) $$\Delta M_{B}=\int_{A}E\left(T_{\mathrm{BTR}}\right)\alpha T_{\mathrm{BTR}}\left(y-\frac{B_{c}}{2}\right)dA$$

(where *y* is the coordinate in the plate width direction, and the neutral axis position is set to $B_{c}/2$). Similarly, the thermally induced restraint resultant force per unit length (corresponding to the membrane component, an equivalent result associated with constraining the thermal eigenstrain) is given by

(15) $$\Delta F_{\mathrm{BTR}}=\int_{A}E\left(T_{\mathrm{BTR}}\right)\alpha T_{\mathrm{BTR}}dA$$

Here, let the “BTR-affected width” be $b_{\mathrm{BTR}}$, and assume that the temperature change is uniform at $T_{\mathrm{BTR}}$ within that range (uniform-BTR-zone approximation). At this time, the cross-sectional area of the zone is $A_{\mathrm{BTR}}=hb_{\mathrm{BTR}}$, and

(16) $$\Delta F_{\mathrm{BTR}}≃hb_{\mathrm{BTR}}E\left(T_{\mathrm{BTR}}\right)\alpha T_{\mathrm{BTR}}$$

Furthermore, if the line of action of the resultant force is regarded as the centroid of the BTR-affected zone $y=b_{\mathrm{BTR}}/2$, the eccentric distance from the neutral axis $y=B_{c}/2$ is $\left(B_{c}/2-b_{\mathrm{BTR}}/4\right)$, so

(17) $$\Delta M_{B}=\left(\frac{B_{c}}{2}-\frac{b_{\mathrm{BTR}}}{4}\right)\Delta F_{\mathrm{BTR}}$$

Next, the relationship between the heat input and the BTR-affected width is given. Letting *Q* be the heat input per unit length on the weld line and assuming a uniform temperature change in the plate thickness direction, the relationship between heat input and $b_{\mathrm{BTR}}$ is given by

(18) $$Q=2c\rho hb_{\mathrm{BTR}}T_{\mathrm{BTR}}$$

where *c* is the specific heat and ρ is the density.

(19) $$I=\frac{hB_{c}^{3}}{12}$$

The curvature κ at the crack initiation location is given by the ratio of bending moment to cross-sectional rigidity. Here, using the room temperature Young’s modulus $E_{\mathrm{RT}}$ as the reference for rigidity evaluation and organizing the temperature dependence as $S=E\left(T_{\mathrm{BTR}}\right)/E_{\mathrm{RT}}$,

(20) $$\kappa =\frac{\Delta M_{B}}{E_{\mathrm{RT}}I}$$

Substituting Equations (16) and (19) into Equation (20) and rearranging,

(21) $$\kappa =\frac{3\alpha S}{2c\rho h}Q\frac{\left(2B_{c}-b_{\mathrm{BTR}}\right)}{B_{c}^{3}}$$

Equation (21) shows that the curvature at the crack initiation location in the side-bead cracking test is uniquely determined by the material properties α,c,ρ, Young’s modulus ratio *S*, heat input *Q*, heated width $b_{\mathrm{BTR}}$, and plate width at the crack arrest position $B_{c}$.

As shown in Figure 11b, when the curvature can be regarded as approximately constant $\kappa =\kappa (B_{c})$ over the BTR section length $L_{\mathrm{BTR}}$, the opening amount at the end of the BTR section can be approximated as

(22) $$\delta_{\mathrm{BTR}}≃\frac{\kappa (B_{c})}{2}L_{\mathrm{BTR}}^{2}$$

Taking $b_{\mathrm{BTR}}$ as the representative width, the representative value of the BTR plastic strain increment is

(23) $$\varepsilon_{\mathrm{BTR}}≃\frac{\delta_{\mathrm{BTR}}}{b_{\mathrm{BTR}}}=\frac{\kappa (B_{c})}{2}\frac{L_{\mathrm{BTR}}^{2}}{b_{\mathrm{BTR}}}$$

Therefore, the crack judgment condition in Section 2 is $\varepsilon_{\mathrm{BTR}}>\varepsilon_{\mathrm{cr}}$, which can be equivalently expressed as

(24) $$\kappa \left(B_{c}\right)>\kappa_{\mathrm{cr}}$$

using the critical curvature $\kappa_{\mathrm{cr}}\equiv 2\varepsilon_{\mathrm{cr}}b_{\mathrm{BTR}}/L_{\mathrm{BTR}}^{2}$. Here, defining $\kappa_{\mathrm{cr}}$ as the critical curvature, the crack arrest width $B_{c}$ in the side-bead cracking test can be interpreted as the intersection point between the curvature–plate width relation and the critical-curvature threshold. Although the present critical-curvature concept is demonstrated for SUS310S under the welding conditions listed in Table 3, the formulation itself is not restricted to this material or process within the side-bead configuration. The material dependence is primarily contained in the cracking threshold, i.e., the critical strain $\varepsilon_{\mathrm{cr}}$ (and, if necessary, the adopted BTR bounds). Therefore, for a different alloy or welding process, the same conversion from $\varepsilon_{\mathrm{cr}}$ to $\kappa_{\mathrm{cr}}$ can be used once $\varepsilon_{\mathrm{cr}}$ is calibrated under the target welding conditions. Conversely, if a side-bead test is conducted for the target material/process and the crack arrest width $B_{c}$ is obtained, $\kappa_{\mathrm{cr}}$ can be back-calculated from $\kappa (B_{c})$ using Equation (21), and the corresponding effective $\varepsilon_{\mathrm{cr}}$ can be obtained via Equation (23). In this way, the proposed framework can be applied to different alloys and welding processes, while the numerical threshold is updated through calibration under consistent conditions.

**Figure 12:**
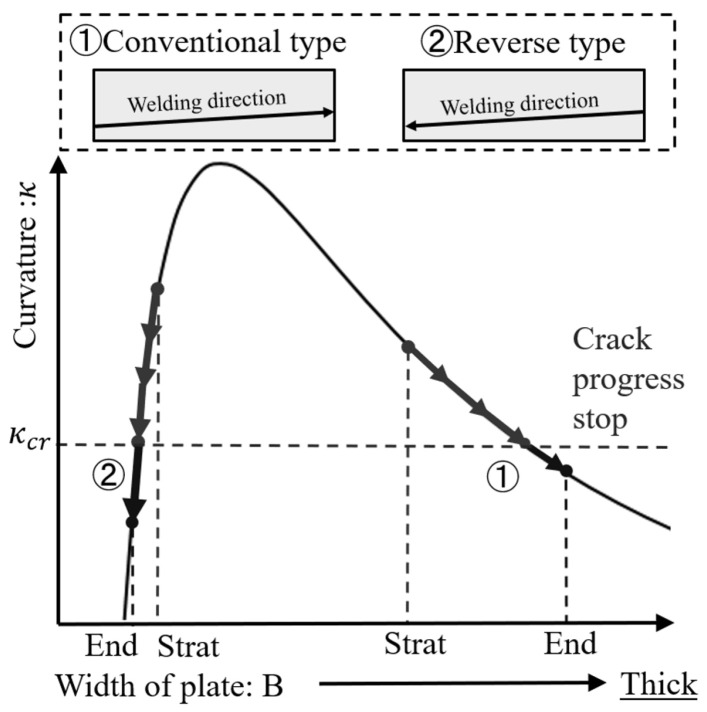
Influence of width of plate on curvature of crack tip.

Using Equation (24), the relationship between plate width and curvature can be organized as shown in Figure 12. The critical curvature is shown as $\kappa_{\mathrm{cr}}$ in the figure as a criterion for crack initiation. In the side-bead cracking test (case ➀ in Figure 12), as welding progresses, the plate width $B_{c}$ near the crack tip increases, so the curvature κ gradually decreases, and it is considered that the crack stops at the position where it falls below the critical curvature. On the other hand, when the welding direction is reversed (case ➁ in Figure 12), the plate width $B_{c}$ near the fusion zone decreases as welding progresses, so the curvature can change steeply. In this case, when $B_{c}$ enters a small region comparable to the heated width $b_{\mathrm{BTR}}$, the contribution of $\left(2B_{c}-b_{\mathrm{BTR}}\right)/B_{c}^{3}$ in Equation (21) becomes relatively large, and the change in curvature becomes pronounced. From the viewpoint of evaluating crack susceptibility by the width $B_{c}$ at the crack arrest position, the condition where the curvature changes gently (as in case ➀) is easier to ensure evaluation accuracy compared to the condition where it changes steeply, as in case ➁. When evaluating by method ➁, it is desirable to set the welding conditions and specimen shape so that the plate width does not change extremely abruptly.

### 4.2. Analysis Model and Analysis Conditions

**Figure 13:**
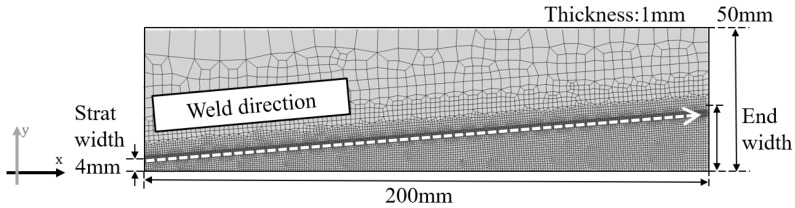
Analysis model.

**Figure 14:**
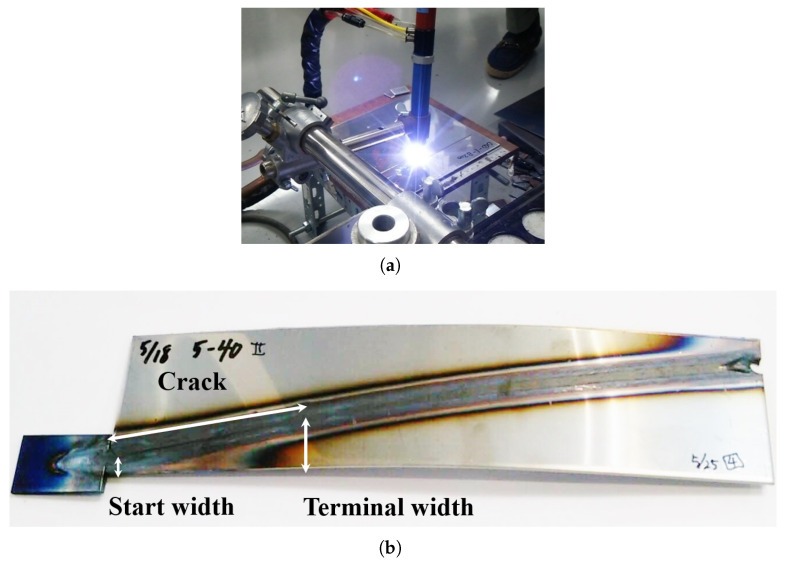
Example of hot cracking in SUS310S(B) side-bead test. (**a**) Welding process. (**b**) Hot crack observed in SUS310(B) side-bead weld.

In this section, the hot cracking analysis method of Maeda [15] used in Section 3 is applied to the side-bead cracking test to verify the validity of the crack initiation, propagation, and arrest model based on the plate width at the crack arrest position. The analysis targets are side-bead test specimens with the same geometry as the mechanical model derived in Section 2, and both Conventional-type and Reverse-type conditions were given the same specimen dimensions and welding conditions as the experiments. Figure 13 shows the geometry and coordinate system of the side-bead test specimen used in the analysis. Figure 14 shows an example of solidification cracks observed in the side-bead cracking test using SUS310S(B). Figure 14a shows the appearance of constricted TIG welding [22]. This process was jointly developed by Tanaka et al. and Murata Welding Laboratory, and unlike general TIG welding, the shield argon gas enables the realization of a bead shape closer to laser welding. Figure 14b shows a typical example of crack arrest behavior targeted in this study, where a crack initiated from the narrow width side propagates along the bead center and stops midway as the plate width increases. The crack arrest position was identified by visual inspection of the weld surface after cooling to room temperature, and the local plate width at the crack tip (measured perpendicular to the weld centerline) was recorded as the crack arrest width. A slight out-of-plane distortion was observed after welding. However, the present side-bead criterion is formulated in terms of the in-plane bending/curvature associated with the local plate width $B_{c}$, and $B_{c}$ is evaluated in the plan view (measured perpendicular to the weld centerline). Therefore, the out-of-plane distortion is regarded as a secondary effect and does not materially affect the evaluation of the crack-arrest width. Representative welding current, voltage, welding speed, heat input efficiency, etc., are shown in Table 3.

**Figure 15:**
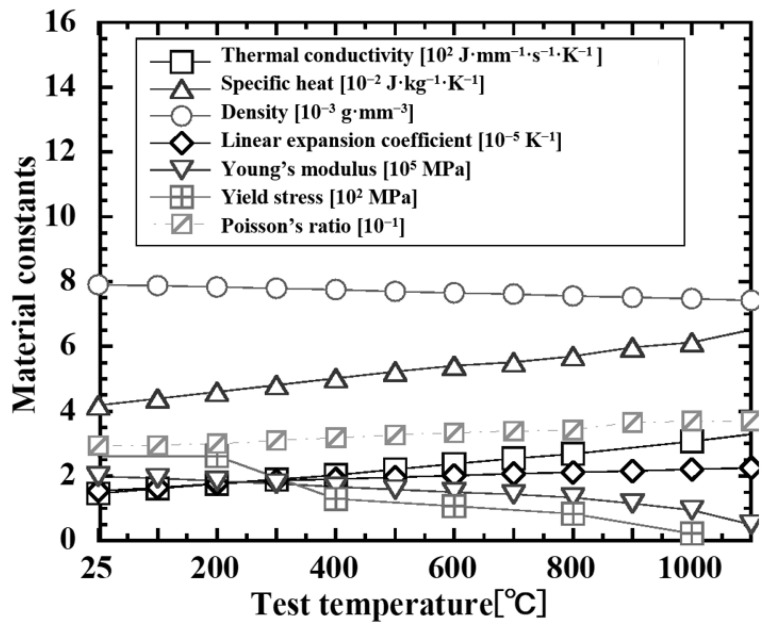
Material constants of stainless steel material, SUS310S(B).

For the analysis, a three-dimensional solid element model was used, and the heat input was applied as a moving line heat source. The material is SUS310S(B), and temperature-dependent properties such as density, specific heat, thermal conductivity, coefficient of linear expansion, Young’s modulus, Poisson’s ratio, and yield stress (defined as the 0.2% proof stress) were used with values shown in Figure 15. The temperature-dependent properties summarized in Figure 15 were taken from the SUS304 dataset in a published high-temperature material-property report/database and used as the analysis input [23], because a complete temperature-dependent property set for SUS310S was not available in a single citable source for this study; therefore, the SUS304 dataset in the published report/database was adopted as a substitute input, noting that elevated-temperature references report broadly comparable magnitudes and temperature-dependent trends of thermo-physical properties and elastic constants across fully austenitic chromium–nickel stainless steels [24]. The BTR was defined as the temperature range between the liquidus temperature and the solidus temperature, and was set to 1320–1440 °C for SUS310S(B). In the present study, different BTR bounds are adopted for the side-bead test and the restrained hot cracking test. This is because, for each test condition, the BTR was defined using the experimentally measured (calibrated) temperature interval specific to that condition. Since the adopted BTR ranges are not identical, caution is required when directly comparing the absolute quantitative values obtained from the two tests. Nevertheless, within each test, the evaluation and discussion are carried out consistently based on the corresponding BTR definition; therefore, the main conclusions of this study and the interpretation of the observed trends are not affected. First, the temperature history of each element was obtained by heat conduction analysis, and then the stress–strain history was calculated by temperature–stress coupled analysis based on the idealized explicit method. The plastic strain during BTR passage was time-integrated for elements near the fusion boundary, and the crack propagation path and arrest position were obtained by extracting the element group that exceeded the set critical strain as the crack propagation region [15].

**Figure 16:**
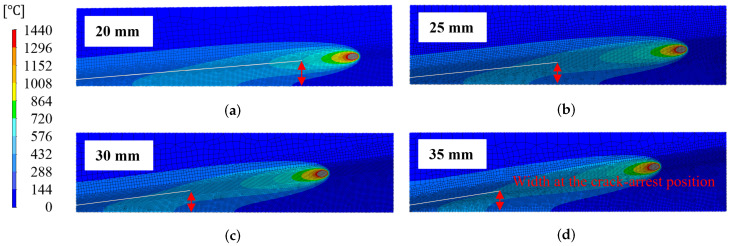
Temperature field and simulated crack-arrest positions in conventional-type side-bead tests; the temperature scale is shown at the left. (**a**) End width 20 mm. (**b**) End width 25 mm. (**c**) End width 30 mm. (**d**) End width 35 mm.

First, the analysis results for the Conventional-type side-bead cracking test are shown in Figure 16. For each condition with the end width changed to 20, 25, 30, and 35 mm, the temperature distribution near the end of welding and the crack propagation region are shown, and the red arrows in the figures correspond to the crack arrest positions in the analysis. As the end width increases, the distance in the welding direction of the crack arrest position increases, while the local plate width at that position converges to approximately 13–16 mm.

**Figure 17:**
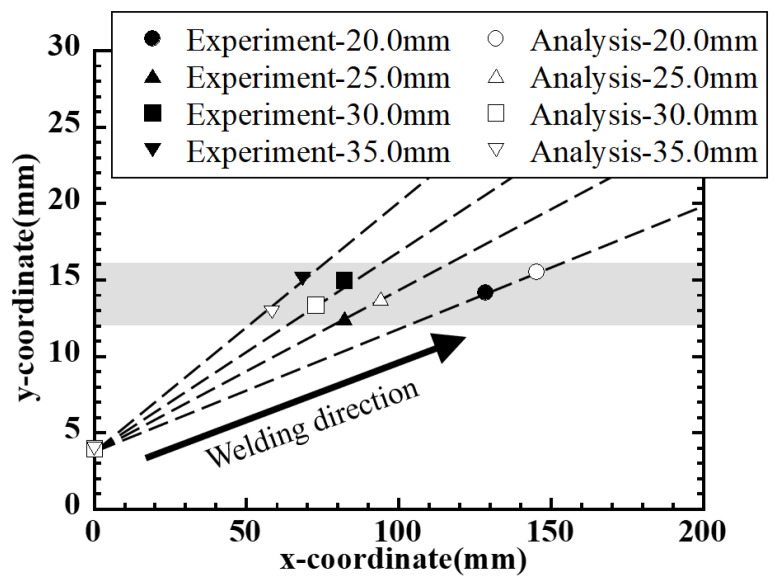
Experimental and geometric crack-arrest position in conventional-type side-bead tests.

A comparison of this tendency with experimental results is shown in Figure 17. The figure shows the coordinates of the crack arrest positions under each condition with end widths of 20–35 mm, where filled symbols represent the crack arrest positions observed in the experiments, and hollow symbols represent analysis values based on the plate width distribution geometrically obtained from the specimen shape. The dashed lines show the plate width distribution B(x) for each condition, and the gray band shows the distribution range of the plate width at the crack arrest position. In both experiments and analysis, the plate width at the crack arrest position is concentrated in a narrow range of approximately 13–16 mm, and the arrest position obtained from numerical analysis is in good agreement with the experimental results.

**Figure 18:**
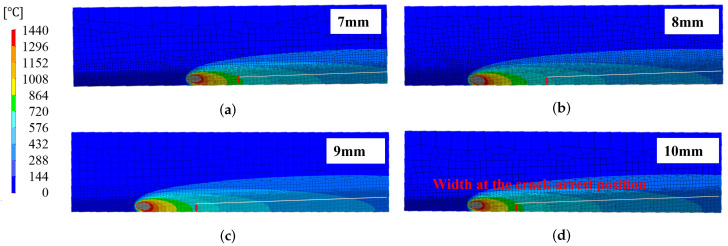
Temperature field and simulated crack-arrest positions in reverse-type side-bead tests; the temperature scale is shown at the left. (**a**) Start width 7 mm. (**b**) Start width 8 mm. (**c**) Start width 9 mm. (**d**) Start width 10 mm.

Next, the analysis results for the Reverse-type side-bead cracking test with the welding direction reversed are shown in Figure 18. In the Reverse type, the end width was kept constant, and the start width was changed to 7, 8, 9, and 10 mm. Each figure shows the temperature distribution near the end of welding and the crack propagation region, and the red arrows correspond to the crack arrest positions in the analysis. Although the crack propagation distance changes depending on the start width, the local plate width at the crack arrest position is concentrated in the range of approximately 4–5 mm in all cases.

**Figure 19:**
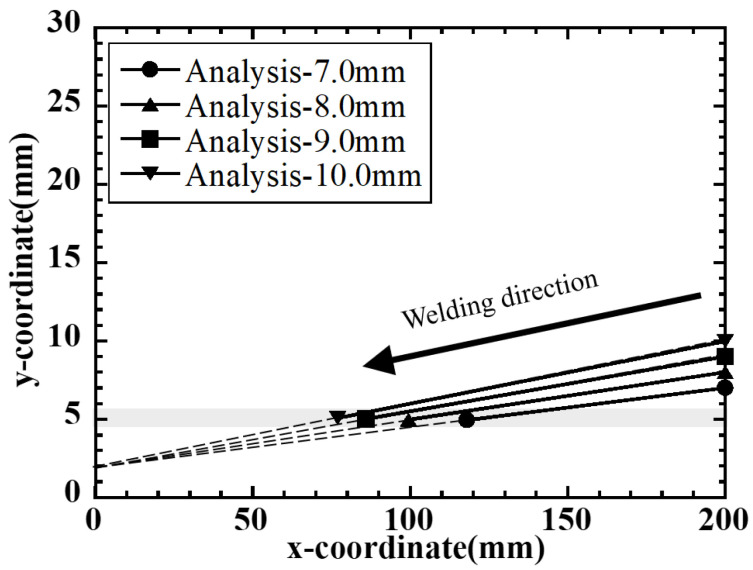
Analysis on crack-arrest positions in reverse-type side-bead tests.

Experimental results for the Reverse type are shown in Figure 19. The solid lines in the figure show the crack propagation path under each condition, the dashed lines show the plate width distribution B(x) obtained from the specimen shape, and the gray band represents the distribution range of the plate width at the crack arrest position. Under all conditions, the crack arrest width converges to approximately 4–5 mm, and although the absolute value differs from 13 to 16 mm in the Conventional type, the crack propagation stops at a certain plate width within the same condition are grouped in all cases.

From the above results, it was confirmed from both experiments and solidification cracking analysis that the crack arrest behavior in the side-bead cracking test is characterized by the local plate width near the crack tip, not by the start width and end width of the entire specimen length, in both the Conventional type and Reverse type. Furthermore, the hot cracking analysis based on the method of Maeda et al. quantitatively reproduced the crack arrest width and arrest position, demonstrating the validity of this analysis method and the modeling using the width at the crack arrest position as an index. In the next section, curvature evaluation based on the mechanical model in Section 4.1 will be performed for these crack arrest widths, and the crack arrest conditions will be organized from the viewpoint of curvature.

### 4.3. Validity Verification of Modeling for Crack Initiation, Propagation, and Arrest Based on Width at Crack Arrest Position

As shown in Section 4.2, the plate width at the crack arrest position is approximately 13–16 mm for the Conventional type and 4–5 mm for the Reverse type, and the absolute value of the crack arrest width differs greatly depending on the welding direction and plate width distribution. On the other hand, using the curvature–plate width relationship derived in Section 4.1, the curvature corresponding to these arrest widths can be evaluated, and the hot cracking arrest condition can be organized as a critical value of curvature.

For each test condition, the local plate width $B_{c}$ at the crack arrest position was read from Figure 16 and Figure 18, and this was substituted into Equation (21) to calculate the curvature κ at crack arrest. For material constants, the representative values of material properties of SUS310S(B) around 1200 °C shown in Figure 15 were used, and for heat input and heated width, the conditions of the side-bead cracking test shown in Table 3 were used.

**Figure 20:**
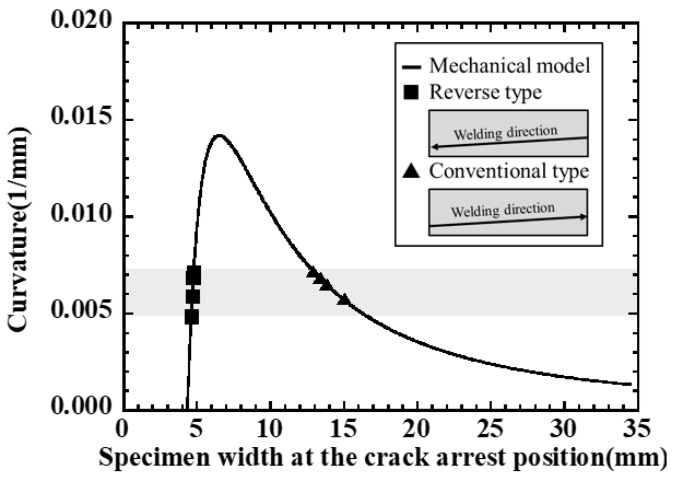
Curvature from mechanical model versus crack-arrest width in conventional and reverse tests.

Figure 20 shows the relationship between curvature and plate width obtained from the mechanical model as solid lines, as well as the curvature obtained from the crack arrest width under each test condition of Conventional type and Reverse type as symbols. From the figure, it can be seen that although the crack arrest width differs between the Conventional type (13–16 mm) and the Reverse type (4–5 mm), the corresponding curvatures are distributed in almost the same narrow range for both. In other words, the crack propagation arrest condition in the side-bead cracking test can be expressed not by the absolute value of the local plate width, but by whether the bending curvature evaluated from the mechanical model for that width reaches a certain critical value. In particular, the critical strain calibrated from the crack-arrest width of the Conventional type is directly used in the Reverse-type evaluation, and the obtained crack-arrest width corresponds to an approximately similar critical-curvature range, indicating that the simplified assumptions have a limited impact on the crack-arrest criterion under the present test conditions.

Because the critical curvature is obtained by rewriting the criterion $\Delta \varepsilon_{\mathrm{BTR}}>\Delta \varepsilon_{\mathrm{cr}}$, the equivalent threshold scales linearly as $\kappa_{\mathrm{cr}}\propto \Delta \varepsilon_{\mathrm{cr}}$. Accordingly, changing $\Delta \varepsilon_{\mathrm{cr}}$ from 0.20 to 0.25% corresponds to a +25% shift in $\kappa_{\mathrm{cr}}$; the curvature range in Figure 20 is κ≈0.005–$0.0075\;{\mathrm{mm}}^{-1}$, and the crack-arrest width remains within the observed window (approximately 13–16 mm).

Since the curvature–plate width curve has a large gradient on the small plate-width side, when the crack-arrest width falls in the several-millimeter region as in the Reverse type, a slight change (or reading uncertainty) in $B_{c}$ is greatly reflected in the converted curvature. In contrast, the arrest width of the Conventional type is located in a relatively gentle region of the curve, and the influence of $B_{c}$ measurement error on curvature evaluation is small; for example, focusing on the forward direction, a reading uncertainty of $\delta B_{c}=1$ mm around $B_{c}\approx 13$–16 mm corresponds to $\delta \kappa \approx 5.0\times 10^{-4}\;{\mathrm{mm}}^{-1}$ on the $\kappa (B_{c})$ curve, which translates (via the curvature-to-strain conversion adopted in this study) to approximately $\delta (\Delta \varepsilon_{\mathrm{cr}})\approx 0.017\%$. From this, in the evaluation of critical curvature using the width at the crack-arrest position, the Reverse type has high sensitivity but tends to have large scatter, while the Conventional type enables more stable evaluation.

From the above, it was shown that the crack arrest behavior in the side-bead cracking test can be organized as a common critical curvature for the results of the Conventional type and Reverse type by evaluating the bending curvature corresponding to the local plate width at the crack arrest position. The mechanical model in Section 4.1 is consistent with three-dimensional finite element analysis and experimental results, and can be said to be an effective simplified index for evaluating solidification cracking susceptibility in the side-bead cracking test.

## 5. Conclusions

In this study, targeting fully austenitic stainless steel, a simplified mechanical model based on plastic strain within the BTR and a mechanical model for the side-bead cracking test were developed with the aim of mechanically evaluating the plate thickness and plate width dependence of hot cracking susceptibility. Furthermore, three-dimensional solidification cracking analysis based on the method of Maeda et al. was applied, and the validity of the proposed models and the effectiveness of the evaluation method based on critical strain and critical curvature were examined through comparison with experimental results. The following findings were obtained:

1. By applying the simplified mechanical model to the restrained hot cracking test, it was shown that the BTR plastic strain increment can be evaluated separately as thermal contraction strain increment, bending strain increment, and restraint strain increment. The non-monotonic plate thickness dependence observed in three-dimensional solidification cracking analysis and experiments was reproduced (“crack susceptibility decreases at intermediate plate thickness and increases on the thin and thick plate sides”), and the mechanical mechanisms were clarified: bending deformation due to temperature gradient in the plate thickness direction is dominant on the thin plate side, and restraint reaction force becomes dominant on the thick plate side.
2. For the side-bead cracking test, a mechanical model was developed focusing on the plate width at the crack arrest position and the corresponding bending curvature. By comparing experimental results and three-dimensional solidification cracking analysis for both Conventional-type and Reverse-type tests, it was shown that the plate width at the crack arrest position converges to an approximately constant value in each condition group even if the specimen shape and welding direction differ, and furthermore that the curvature corresponding to that width can be organized as a common critical curvature, demonstrating the mechanical significance of crack susceptibility evaluation using the width at the crack arrest position.
3. By using the simplified mechanical model and curvature evaluation equation, it was shown that hot cracking susceptibility for combinations of plate thickness, plate width, and heat input conditions can be easily evaluated based on material-specific critical strain or critical curvature. Beyond serving as an evaluation index, the proposed model suggests that selecting plate thickness, heat input, and restraint conditions to reduce the tensile plastic strain increment accumulated within the BTR can be an effective route to mitigate hot cracking. Since critical strain and critical curvature are quantities that depend on welding conditions, it was clarified that when comparing crack susceptibility between materials, it is necessary to conduct tests under the same welding conditions and evaluate the width at the crack arrest position as a consistent index.

## Figures and Tables

**Table 1:** Chemical composition of material used.

C (wt.%)	Si (wt.%)	Mn (wt.%)	P (wt.%)	S (wt.%)	Cr (wt.%)	Ni (wt.%)	N (wt.%)	O (wt.%)
0.001	0.1	0.8	0.1	0.001	24.5	21.5	0.001	0.005

**Table 2:** Welding conditions.

Current *I* (A)	Arc Voltage *V* (V)	Welding Speed *v* (mm/s)	Heat Efficiency η
150	15	1.66	0.7

**Table 3:** Welding conditions for side-bead cracking test.

Welding Conditions					
Welding current	Welding voltage	Welding speed	Start width	End width	Heat input per unit length
*I* (A)	*V* (V)	*v* (mm/min)	$b_{\mathrm{start}}$ (mm)	$b_{\mathrm{end}}$ (mm)	*Q* (J/mm)
82	12	450	4	variable	131.2

## Data Availability

The original contributions presented in this study are included in the article. Further inquiries can be directed to the corresponding author.
